# Improved detection of *Pneumocystis jirovecii *in upper and lower respiratory tract specimens from children with suspected pneumocystis pneumonia using real-time PCR: a prospective study

**DOI:** 10.1186/1471-2334-11-329

**Published:** 2011-11-28

**Authors:** Catherine M Samuel, Andrew Whitelaw, Craig Corcoran, Brenda Morrow, Nei-Yuan Hsiao, Marco Zampoli, Heather J Zar

**Affiliations:** 1 Division of Medical Microbiology, University of Cape Town and National Health Laboratory Service, Cape Town, South Africa; 2 Division of Clinical Virology, University of Cape Town and National Health Laboratory Service, Cape Town, South Africa; 3 Department of Paediatrics and Child Health, University of Cape Town, Red Cross War Memorial Children's Hospital, Cape Town, South Africa

## Abstract

**Background:**

*Pneumocystis *pneumonia (PCP) is a major cause of hospitalization and mortality in HIV-infected African children. Microbiologic diagnosis relies predominantly on silver or immunofluorescent staining of a lower respiratory tract (LRT) specimens which are difficult to obtain in children. Diagnosis on upper respiratory tract (URT) specimens using PCR has been reported useful in adults, but data in children are limited. The main objectives of the study was (1) to compare the diagnostic yield of PCR with immunofluorescence (IF) and (2) to investigate the usefulness of upper compared to lower respiratory tract samples for diagnosing PCP in children.

**Methods:**

Children hospitalised at an academic hospital with suspected PCP were prospectively enrolled. An upper respiratory sample (nasopharyngeal aspirate, NPA) and a lower respiratory sample (induced sputum, IS or bronchoalveolar lavage, BAL) were submitted for real-time PCR and direct IF for the detection of *Pneumocystis jirovecii*. A control group of children with viral lower respiratory tract infections were investigated with PCR for PCP.

**Results:**

202 children (median age 3.3 [inter-quartile range, IQR 2.2 - 4.6] months) were enrolled. The overall detection rate by PCR was higher than by IF [180/349 (52%) vs. 26/349 (7%) respectively; p < 0.0001]. PCR detected more infections compared to IF in lower respiratory tract samples [93/166 (56%) vs. 22/166 (13%); p < 0.0001] and in NPAs [87/183 (48%) vs. 4/183 (2%); p < 0.0001]. Detection rates by PCR on upper (87/183; 48%) compared with lower respiratory tract samples (93/166; 56%) were similar (OR, 0.71; 95% CI, 0.46 - 1.11). Only 2/30 (6.6%) controls were PCR positive.

**Conclusion:**

Real-time PCR is more sensitive than IF for the detection of *P. jirovecii *in children with PCP. NPA samples may be used for diagnostic purposes when PCR is utilised. Wider implementation of PCR on NPA samples is warranted for diagnosing PCP in children.

## Background

*Pneumocystis *pneumonia (PCP), caused by *Pneumocystis jirovecii*, is an important opportunistic infection in HIV-infected children [1,2]. The incidence of PCP in developed countries has declined since the introduction of highly active anti-retroviral therapy and use of chemoprophylaxis. However, PCP remains a major cause of hospitalization and mortality in HIV-infected children in low or middle income countries, [1,3-5] with reported incidence rates of 10 - 49%, [1,3,6] and in-hospital case-fatality rates of 20 - 63% [1,3,4,6]. Apart from HIV infection, there are other factors that predispose children to developing PCP including malnutrition, other immune deficiencies or HIV exposure. Untreated, the case fatality rate in children with PCP approximates 100% [1,3,4,6]. However, diagnosis can be difficult as clinical and radiological findings are non-specific. Therefore, a rapid, accurate laboratory diagnosis is important for timely use of appropriate medication.

Detection of *P. jirovecii *is hampered by the lack of a sustainable *in-vitro *culture method [7]. Standard laboratory diagnostic methods are microscopic examination of a lower respiratory tract sample with Gomori Grocott's methenamine silver nitrate stain or the more sensitive immunofluorescence assay (IF) [8] on bronchoalveolar lavage (BAL) or induced sputum (IS) specimens. Using microscopy, the yield from IS has been reported to be similar compared to that from BAL [9]. However, sputum induction in children is not widely performed, requires staff trained to do the procedure and may result in clinical deterioration or nosocomial transmission of respiratory pathogens. Diagnosis using a non-invasive sample such as a nasopharyngeal aspirate (NPA) is, therefore, desirable.

The clinical sensitivity from a NPA has been reported to be low and variable when microscopy is utilised [4,5,10]. The development of polymerase chain reaction (PCR) techniques has provided a more reliable diagnostic method. In adult studies, PCR is as specific as and more sensitive than microscopy for diagnosis when performed on respiratory specimens, including oral washes [7,11-21]. In a study of oropharyngeal washes from HIV-infected adult patients, *P. jirovecii *DNA-amplification had a sensitivity of 44% using a nested PCR protocol compared to trans-bronchial biopsy, [16] increasing to 90% when touch-down real-time PCR was utilised [7,11]. Real-time PCR also allows for quantification of the organism load and with application of cutoff values, could improve the specificity by distinguishing between colonization and infection [13].

The aims of this study were (1) to compare a real-time quantitative PCR assay with IF for the diagnosis of PCP in children and (2) to evaluate the reliability of PCR for the diagnosis of PCP in upper compared to lower respiratory tract secretions.

## Methods

### Participants

Consecutive children (<14 years old) with suspected PCP, hospitalized at Red Cross War Memorial Children's Hospital, Cape Town, South Africa, were enrolled from November 2006 to August 2008. Clinical criteria for suspected PCP were an acute onset of a respiratory illness, presence of age-specific tachypnoea and hypoxia, bilateral lung disease (not associated with wheezing) and a risk factor for PCP (HIV-infected, HIV-exposed, malnourished, receiving immunosuppressive therapy or immunodeficiency disease other than HIV). Patients were excluded if they had received treatment for PCP in the preceding 2 weeks or were on PCP treatment for more than 48 hours. A child was defined as HIV-infected if they had a positive HIV PCR (Amplicor HIV-1 DNA test version 1.5, Roche Diagnostics GmbH, Mannheim, Germany) if younger than 18 months or a positive HIV ELISA (Architect HIV Ag/Ab Combo ELISA, Abbott Laboratories, Abbott Park, IL) in older children. HIV exposure in infants less than 18 months was defined as being HIV sero-positive with a negative HIV PCR. Children were treated according to a standard protocol for severe pneumonia that included intravenous co-trimoxazole and oral corticosteroids, as well as broad-spectrum antibiotics. Other antimicrobial therapy was added at the discretion of the attending clinician. Written informed consent was obtained from a parent or legal guardian. The study was approved by the Research Ethics Committee of the Faculty of Health Sciences at the University of Cape Town.

### Sample collection

An upper respiratory tract sample (NPA) and a lower respiratory tract sample (IS in non-intubated patients or BAL in intubated patients) were obtained using standardised methods at the time of admission [11,22]. NPA specimens were obtained first, followed by LRT specimens. After routine laboratory investigations had been performed, an aliquot of sample was frozen at -70°C for analysis by PCR.

### Control group

Respiratory samples (NPA or BAL) from 30 children sequentially hospitalized with a confirmed viral LRT infection, who were randomly selected and who improved clinically without specific treatment for PCP, were investigated with PCR for PCP, as a control group.

### Laboratory investigations

Direct immunofluorescence (IF) (Detect IF PC, Axis-Shield Diagnostics, Cambridgeshire, United Kingdom), to detect cystic forms of *P. jirovecii *using a monoclonal antibody immunofluorescent stain, was performed according to the manufacturer's instructions. Accordingly, results were reported as positive if more than 5 cysts were observed. In addition, Grocott's methenamine silver nitrate stain was performed on BAL specimens, as per clinician's request, if sufficient sample volume was available.

DNA was isolated using the Nuclisens EasyMAG platform (bioMérieux, Boxtel, Netherlands). *P. jirovecii *DNA was detected using a quantitative, touch-down, real-time PCR assay, targeting the major surface glycoprotein (MSG) gene as described by Larsen *et al *[13]. Commercially synthesized primers, amplifying a 250-bp segment of the multicopy MSG gene family, were utilized in a PCR reaction containing fluorescence resonance energy transfer (FRET) probes for detection of the amplified product [13]. The *P. jirovecii *MSG gene was cloned into the pCR 2.1 vector. Four external standards of the cloned target template, corresponding to 10^6^, 10^5^, 10^4 ^and 10^3 ^copies per microlitre, were used in each PCR run to generate an external standard curve, required for quantification. The reactions were performed on the LightCycler platform (Roche Diagnostics GmbH, Mannheim, Germany) and results were calculated using the LightCycler software and expressed in copies/mL. A *P. jirovecii *positive and a negative control were included in each PCR run. Laboratory testing was conducted in an ISO-accredited molecular laboratory of the National Health Laboratory Service (NHLS) at Groote Schuur Hospital, Cape Town. The laboratory is designed with separate areas for DNA extraction, PCR preparation, DNA addition, and amplification, in order to reduce the chance of cross contamination. Use of a real-time PCR platform minimised the chance of contamination during the detection step. The investigators who performed the PCR testing were blinded to the IF or silver stain results.

### Data Analysis

Statistical analysis was performed using statistical software Stata (version 10.0, StataCorp, Houston, USA). Continuous data was tested for normality using the Shapiro-Wilk test. Detection rates of PCR were compared by Pearson's chi-squared test and quantification results were compared by Wilcoxon rank-sum (Mann-Whitney) test. Paired analysis of continuous and binary variables was performed using t test and chi-squared test, respectively.

## Results

### Patient characteristics

212 children were enrolled (Table 1); 10 patients were excluded as there was insufficient respiratory sample left for PCR, thus 202 children were included in this analysis. Of these, 92 (46%) were male; the median (IQR) age was 3.3 (2.2 - 4.6) months. HIV status was determined in 200 (99%) patients, of whom 129 (65%) were HIV-infected. Of the 71 (35%) HIV-uninfected children, 32 (45%) were HIV-exposed. Twenty-seven (21%) of the 129 HIV-infected children were on co-trimoxazole prophylaxis at presentation. The median (IQR) CD4 percentage of HIV-infected children was 16.9% (10.1 - 27.1)

**Table 1:** Baseline characteristics of children admitted to hospital with suspected *Pneumocystis jirovecii *pneumonia

Characteristic	All Patients (n = 202)	Patients with positive PCR for PCP (n = 110)
**Male**	92 (46%)	49 (45%)
**Median age, IQR, months**	3.3 (2.2-4.6)	3.4 (2.7-3.9)
**HIV positive**	129/200 (65%)	92/109 (84%)
Median CD4 percentage*, IQR, %	16.9 (10.1-27.1)	13.6 (9.0-18.0)
Cotrimoxazole prophylaxis*	27 (21%)	10 11%)

* In HIV-infected children

### PCR and IF on patient samples

349 respiratory samples were obtained. One hundred and forty-seven (73%) children had paired samples consisting of a NPA with either a BAL or IS. Fifty-five children produced a single respiratory sample (Table 2).

**Table 2:** Sources of the 349 samples from 202 patients included in the study

	Unpaired Samples	Paired Samples		Total Samples
		NPA+IS	NPA+BAL	
**NPA**	36	92	55	183
**IS**	8	92	-	100
**BAL**	11	-	55	66
**Total Samples**	55	184	110	349

URT, upper respiratory tract; LRT, lower respiratory tract; NPA, nasopharyngeal aspirate; IS, induced sputum; BAL, bronchoalveolar lavage

Real-time PCR performed on 349 respiratory samples detected *P. jirovecii *in 180 specimens (52%) (Table 3). Of the 202 patients, 110 (54.5%) had one or more specimens positive by PCR. The HIV status was determined in 109 of these 110 patients. 92 (84%) of the 109 patients with a positive PCR result were HIV infected. This was significantly higher than in the total cohort of patients where 65% were found to be HIV-infected (Odds-ratio OR, 7.9; 95% CI 3.9-16.3; p < 0.0001).

**Table 3:** Quantitative PCR and Direct IF performed on 349 respiratory samples collected from 202 patients

	TOTAL	URT	LRT	OR (95% CI)
	(n = 349)	(n = 183)	(n = 166)	
**Positive PCR**	180 (52%)	87 (48%)	93 (56%)	0.71 (0.46 - 1.11)
**Positive IF**	26 (7%)	4 (2%)	22 (13%)	0.15 (0.04 - 0.45)

URT, upper respiratory tract; LRT, lower respiratory tract; OR, Odds-ratio; CI, confidence interval; PCR, polymerase chain reaction; IF, immunofluorescence

Overall, *P. jirovecii *DNA was detected in 37 of 66 (56%) BAL specimens, 56 of 100 (56%) IS and 87 of 183 (48%) NPA. There was no significant difference between the detection by PCR on LRT samples compared to NPA (OR, 0.71; 95% CI, 0.46 - 1.11; Table 3).

In contrast, *P. jirovecii *was detected by IF in 26 (7%) of samples, consisting of 13 (50%) BAL, 9 (35%) IS and 4 (15%) NPA samples. All 26 IF-positive samples were positive on PCR and thus no additional cases were detected using IF. The overall yield on upper tract samples was 48% (87/183) by PCR, as compared to the yield of 2% (4/183) by IF (OR 40.5; 95% CI, 14.4-113.8; p < 0.0001). For lower tract samples, the yield from PCR was 56% (93/166) compared to 13% (22/166) by IF (OR 8.3; 95% CI, 4.8-14.3; p < 0.0001).

*P. jirovecii *was also demonstrated on silver stain in 8 of the 25 (32%) BAL samples sent for analysis. All 8 were also PCR positive, and 3 were IF positive. Of the 17 patients with a negative BAL silver stain, 5 (29%) were PCR-positive and 3 were also IF positive.

### Comparison of yield by PCR on paired upper and lower respiratory tract samples

Of the 147 paired upper and lower respiratory tract samples, 70 pairs were concordant PCR-positive, 63 pairs were concordant PCR-negative and 14 pairs had discordant PCR results. Of the discordant pairs, most were PCR positive on the lower tract sample but negative on the upper tract sample (Table 4). Overall, the detection from upper and lower specimens by PCR was similar with 81 of 147 (55%) lower respiratory tract samples positive by PCR vs. 73 of 147 (50%) NPA samples (OR, 1.4; 95% CI, 0.91-2.19). Only 8 (5%) additional cases were detected by PCR when performed on a lower respiratory tract sample compared to a NPA specimen (p = 0.11). Using a positive PCR result on a lower respiratory tract sample as a gold standard, the sensitivity, specificity, positive predictive value and negative predictive value for PCR on a NPA sample was 86%, 95%, 96% and 85% respectively.

**Table 4:** Real-time PCR results on paired upper and lower respiratory tract samples (n = 147)

	LRT Sample	LRT Sample	Total
	(IS/BAL) Positive	(IS/BAL) Negative	
**URT Sample (NPA) Positive**	70 (48%)	3 (2%)	73 (50%)
**URT Sample (NPA) Negative**	11 (7%)	63 (43%)	74 (50%)
**Total**	81	66	147

URT, upper respiratory tract; LRT, lower respiratory tract; NPA, nasopharyngeal aspirate; IS, induced sputum; BAL, bronchoalveolar lavage

Concentrations of *P. jirovecii *DNA ranged from 3.2 to 9.4 log copies/mL. The median (IQR) organism load detected in NPA samples was significantly lower than that detected in lower respiratory tract samples [5.9 (5.4 - 6.7) log copies/ml compared to 6.6 (5.8 - 7.5) log copies/mL; p = 0.0002).

### Control group

Of the 30 children in the control group, 18 (60%) were male; the median (IQR) age was 6.0 (4.0 - 12.0) months. HIV status was determined in 21 (70%) patients, of whom 2 (10%) were HIV-infected, 6 (28%) were HIV-exposed and 13 (62%) were HIV-unexposed. Twenty-eight (93%) of 30 control samples were PCR-negative. The 2 PCR positive samples (both NPA samples), were from HIV-unexposed infants, younger than 6 months old, with DNA concentrations of 6.1 and 6.7 log copies/mL.

## Discussion

This study found that PCR had a much higher sensitivity than the current standard diagnostic test of IF, with a more than 5 fold increased detection in upper and lower samples using PCR. In addition, an URT specimen was as reliable as a LRT specimen for PCR confirmed diagnosis. To our knowledge, this is the first study in children that demonstrates that *P. jirovecii *can be reliably detected using PCR on an upper respiratory tract specimen. The increase in diagnostic yield using PCR occurred in both upper and LRT specimens, but was especially marked in upper respiratory samples. This is important as obtaining a LRT specimen using BAL or IS in children can be difficult whereas obtaining a NPA is much easier, and carries a lower risk of nosocomial transmission of infection, thus making the diagnosis of PCP feasible in many health-care settings, including in primary care. The detection of organism in an URT sample may occur due to extension of infection to the upper airways in severe disease or by organisms that are propelled to the upper airway during coughing [16].

Using current methods of detection with staining, PCP may therefore be substantially under-diagnosed in children. This is supported by the low rates of laboratory confirmation of approximately 20% reported in clinical studies of children with suspected PCP [1,3]. When IF on LRT samples was used as the reference standard, the sensitivity of the PCR assay was 100%.

A further advantage of PCR is the rapid turn-around time of the test, with results obtainable within hours. Children with PCP often have a rapidly progressive course, thus timely diagnosis would allow early initiation of treatment in those not on treatment, with potential reduction in mortality. The usual management of suspected PCP is empiric treatment with co-trimoxazole and corticosteroids in children who are hypoxic. Rapid confirmation of diagnosis would therefore also be useful for guiding corticosteroid therapy. This is especially relevant given the concerns about use of corticosteroids in other infections such as CMV pneumonitis or TB in children, without specific antimicrobial therapy for these pathogens [23,24]. The clinician may also consider discontinuation of co-trimoxazole therapy in patients with negative PCR results and contemplate alternative diagnoses.

The specificity of PCR for PCP is more difficult to determine as the organism cannot be cultured and there is no method to distinguish disease from asymptomatic colonization. The large number of IF-negative, PCR-positive samples may therefore represent children with PCP or with pneumocystis colonization. As all children were treated for PCP based on their clinical presentation, it was impossible to use response to therapy to distinguish active disease from colonization. The organism has been reported to colonize the airways of adults with chronic obstructive airway disease, with these individuals forming the reservoir for infection of susceptible hosts [25]. Moreover, positive PCR results have been reported in BAL samples from adults without clinical PCP, HIV, or other infections [26]. However, all children in the study had an underlying risk factor for PCP and were severely ill with a clinical presentation consistent with PCP. In addition most children were under 6 months of age, the highest risk period for primary PCP rather than colonization. It is, therefore, likely that a positive PCR result in these children represents disease.

To further investigate the specificity of PCR for PCP samples from 30 control children with confirmed viral LRT illness who improved clinically without therapy for PCP, were tested. In contrast to the detection rate in cases (110, 55%), only a minority (2, 6%) of control children were PCR positive. The 2 control positives may represent primary infection which is very common in children in the first years of life, and may manifest as a viral-like illness. Another possible explanation may be laboratory contamination. However, this is unlikely as the laboratory layout allows only unidirectional work-flow with strict separation of all steps in the PCR process. The real-time PCR was performed in a closed system, with detection occurring on the same platform thus minimizing the risk of contamination.

An alternative strategy for differentiating colonization from disease is by applying cut-off values to quantitative PCR results. Huggett *et al *were able to achieve a specificity of 96% in BAL specimens from adult patients by using a quantitative PCR for HSP70, with appropriate cut-off values [27]. Unfortunately, the quantitative results obtained in our study could not be accurately interpreted as data was not normalized by use of a reference gene as an internal control. Normalization may control for variations in the extraction process, patient variability and inter-sample variability by accounting for the dilution factor in the BAL fluid. However, there is controversy about the choice of the most appropriate reference gene [28]. If normalization can be reliably performed, it may allow for more accurate estimation of colonization rates and of cut-off values to distinguish disease thus improving the specificity of the assay. Further work in this area is needed.

Potential difficulties in achieving widespread implementation of PCR for PCP diagnosis include cost and the need for a specialized molecular laboratory, equipment, and personnel. However, much progress has been made in developing closed molecular amplification platforms for the diagnosis of TB at point-of-care facilities. Given the scale of the HIV pandemic and the substantial mortality associated with PCP particularly in children, a similar approach for the diagnosis of PCP, should be undertaken.

## Conclusion

In summary, this real-time PCR-based assay has a much higher sensitivity than current diagnostic tests and allows for the use of a NPA sample for reliable detection of *P. jirovecii *in children. PCR on a NPA should replace the traditional diagnostic method of IF or silver stain performed on LRT samples. The possibility of making a reliable diagnosis on a NPA represents an important advance in confirming PCP in children. Widespread implementation of this technology is needed especially in high burden HIV and resource limited settings.

## Competing interests

The authors declare that they have no competing interests.

## Authors' contributions

HZ was responsible for study conception and design, clinical supervision and obtained funding. AW was the laboratory supervisor, contributed to the study design and obtained funding. CC assisted in setting up the molecular assay. BM and MZ recruited patients and acquired data. MH assisted in the statistical analysis. CS was the project leader in the laboratory, performed the molecular investigations and is first author of the manuscript. All authors contributed to the final manuscript and have read and approved of it.

## Pre-publication history

The pre-publication history for this paper can be accessed here:

http://www.biomedcentral.com/1471-2334/11/329/prepub
